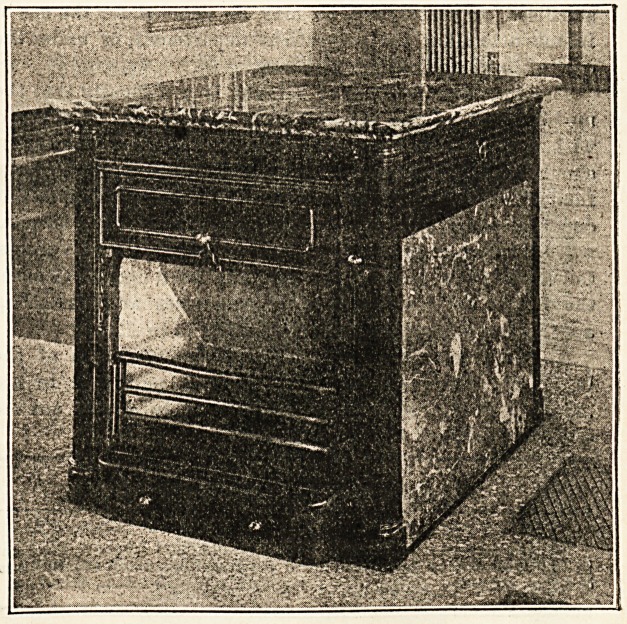# Practical Departments

**Published:** 1904-02-20

**Authors:** 


### PRACTICAL DEPARTMENTS.

### HEATING.

THE fireplace illustrated in this week's issue is D. O. Boyd's "Hygiastic," pattern "B," manufactured by Messrs. Hendry and Patterson, Ltd. One of the great advantages of a stove of this type is that it can be placed in the middle of a ward and yet not obstruct the nurse's vision. The stove illustrated is double and has separate flues. The casing is in marble in either "Rouge Royal" or "St. Ann's," and the side panels are made to open so that the flues can be swept from the interior of the stove. There is a special balance blower, and fresh air is let into the space between, and around the steel flues from which it escapes into the ward by special regulation valves. Some authorities are strong upon having fire-clay air flues instead of metal flues but one of the points the makers claim is that in this case the air does not get burnt. Upon the removal of the side panels in the air chamber there are seen small cast-iron panels in the sides of the flues fixed with thumb-screws and these, the makers state, should be well puttied before fixing We would suggest to architects and others responsible for carrying out the work that these stoves should be specified "to be fixed complete and tested by the makers." And to the makers that they should employ only their best men for this kind of work. Otherwise there is a great risk (if not properly set) of the fresh air flues being contaminated by the smoke or other impurities. In our illustration horizontal bars are shown but these are <-shaped and their upper faces slope inwards to throw off the cinders, but the makers will supply vertical bars if required, which are preferable.

The makers also supply a small collecting pan for ashes falling from the front of the fire, but one extending right underneath would be better, for few neurotic invalids appreciate the scraping of a shovel upon the hearth. It is the practice of Messrs. Hendry and Co. not to make use of the top of their stoves for heating purposes, and instead, they protect the under side of their handsome 2 in. slabs of marble with asbestos or slag wool carried upon sheet steel and "tee" irons, so that the top may be used for standing plants, etc., upon. In use these stoves are said to be very economical and they are certainly ornamental without being ornate, and the makers are justly proud of the long list of municipal and other authorities whom they have supplied.

Messrs. Hendry and Co. have brought out a further improvement which is said to do away with the necessity of lighting a pilot fire in order to start the draw, these are known as pattern "C" and have no fire-bars.

BRITISH THOMSON-HOUSTON ELECTRIC LAMPS.

We have recently tested the durability and general efficiency of these lamps and are satisfied that they are of first-rate quality. An electric lamp, however cheap, which cannot be relied upon is an expensive article, but as the British Thomson-Houston lamps are of moderate price and undoubted durability, they may confidently be recommended; and moreover the company is prepared to enter into yearly contracts for the supply of their lamps to institutions.

## Figures and Tables

**Figure f1:**